# Direct Oral Anticoagulants, COX-2–Selective NSAIDs, and Gastrointestinal Bleeding in Atrial Fibrillation

**DOI:** 10.1001/jamanetworkopen.2026.13941

**Published:** 2026-05-26

**Authors:** Fabian Maximilian Meinert, Jenny Dimakos, Ying Cui, Kristian B. Filion, Christel Renoux, Antonios Douros

**Affiliations:** 1 Institute of Clinical Pharmacology and Toxicology, Charité-Universitätsmedizin Berlin, Berlin, Germany; 2 Department of Medicine, McGill University, Montreal, Quebec, Canada; 3 Centre for Clinical Epidemiology, Lady Davis Institute, Montreal, Quebec, Canada; 4 Department of Epidemiology, Biostatistics and Occupational Health, McGill University, Montreal, Quebec, Canada; 5 Department of Neurology and Neurosurgery, McGill University, Montreal, Quebec, Canada

## Abstract

**Importance:**

It is unclear whether the beneficial effects of nonsteroidal anti-inflammatory drugs (NSAIDs) that selectively inhibit cyclooxygenase 2 (COX-2) in decreasing the risk of gastrointestinal (GI) bleeding are retained in patients with nonvalvular atrial fibrillation (NVAF) concomitantly treated with direct oral anticoagulants (DOACs).

**Objective:**

To assess whether concomitant use of DOACs and COX-2–selective NSAIDs is associated with a decreased risk of GI bleeding vs concomitant use of DOACs and nonselective NSAIDs in patients with NVAF.

**Design, Setting, and Participants:**

This multinational cohort study used electronic medical records from the UK and claims data from Quebec between January 1, 2011, and December 12, 2020. Adult patients (aged ≥18 years) with NVAF who initiated concomitant use of DOACs and NSAIDs during the study period were included. Cohort entry was the first day of concomitant use. Patient data were followed until a study outcome, death, or treatment discontinuation or switch. All analyses were conducted between November 19, 2024, and July 25, 2025.

**Exposures:**

Concomitant use of DOACs and COX-2–selective NSAIDs or concomitant use of DOACs and nonselective NSAIDs.

**Main Outcomes and Measures:**

The primary outcome was GI bleeding, and the secondary outcome was non-GI bleeding. Inverse probability of treatment weighting was used to control for confounding. Cox models were used to estimate site-specific hazard ratios (HRs) and 95% CIs, which were pooled using random-effects models. Further stratification was performed for potential effect modifiers.

**Results:**

The study cohort included 30 240 patients with NVAF who initiated concomitant use of DOACs and NSAIDs (mean [SD] age, 72.1 [9.2] years, 17 146 male [56.7%]). They contributed 37 833 episodes of concomitant use of DOACs and NSAIDs (45.2% COX-2–selective NSAIDs, 54.8% nonselective NSAIDs). Concomitant use of DOACs and COX-2–selective NSAIDs was associated with a decreased risk of GI bleeding (pooled weighted HR, 0.63 [95% CI, 0.46-0.87]; *I*^2^ = 56%) and non-GI bleeding (pooled weighted HR, 0.54 [95% CI, 0.40-0.74]; *I*^2^ = 0%) vs concomitant use of DOACs and nonselective NSAIDs. Female patients had a greater decreased risk of GI bleeding associated with concomitant use of DOACs and COX-2–selective NSAIDs (pooled weighted HR, 0.50 [95% CI, 0.31-0.80]; *I*^2^ = 0%) than male patients (HR, 0.85 [95% CI, 0.55-1.32]; *I*^2^ = 78%). There was no major effect measure modification by age, order of drug initiation in concomitant use, baseline bleeding risk, or individual DOACs.

**Conclusions and Relevance:**

These findings suggest that COX-2–selective NSAIDs may retain their beneficial effects regarding GI bleeding during concomitant use with DOACs. Future studies should examine the association of COX-2 selectivity with stroke risk among patients treated with DOACs.

## Key Points

**Question:**

Is there a difference in the risk of gastrointestinal (GI) bleeding between cyclooxygenase 2 (COX-2)–selective and nonselective nonsteroidal anti-inflammatory drugs (NSAIDs) among patients treated with direct oral anticoagulants (DOACs)?

**Findings:**

In this cohort study of 30 240 patients with nonvalvular atrial fibrillation, concomitant use of DOACs and COX-2–selective NSAIDs was associated with a statistically significant 37% decrease in the risk of GI bleeding vs concomitant use of DOACs and nonselective NSAIDs.

**Meaning:**

These findings suggest that COX-2–selective NSAIDs may retain their beneficial effects regarding GI bleeding during concomitant use with DOACs.

## Teaser

This cohort study assesses whether concomitant use of direct oral anticoagulants and cyclooxygenase 2 (COX-2)–selective nonsteroidal anti-inflammatory drugs (NSAIDs) is associated with a decreased gastrointestinal bleeding risk among patients with nonvalvular atrial fibrillation.

## Introduction

Nonsteroidal anti-inflammatory drugs (NSAIDs) are commonly used analgesics that exert their effects through the inhibition of the cyclooxygenase 1 (COX-1) and cyclooxygenase 2 (COX-2) enzymes. Nonsteroidal anti-inflammatory drugs are known for their propensity to cause adverse effects in the gastrointestinal (GI) tract, including mild conditions, such as indigestion, and more severe reactions, such as gastroduodenal ulcerations and potentially GI bleeding.^1^ Indeed, NSAIDs have been shown to increase the risk of GI bleeding up to 4 times compared with nonuse.^2^

In the 1990s, NSAIDs that selectively inhibit COX-2 (eg, celecoxib) were developed with the aim, among other things, of reducing the risk of GI bleeding.^3^ The rationale was based on the pivotal role of COX-1 in the building of prostaglandins that protect the gastric mucosa from ulcerating; therefore, selective inhibition of COX-2 would interfere with this protection to a lesser degree. Indeed, subsequent randomized clinical trials corroborated these pharmacologic considerations, showing that the use of COX-2–selective NSAIDs lead to an up to 60% decrease in the risk of GI bleeding compared with the use of nonselective NSAIDs.^4,5,6^

While the beneficial effects of COX-2 selectivity for NSAID-related GI bleeding are clear among patients with indications for analgesic treatment, such as rheumatoid arthritis or osteoarthritis,^4,5,6^ there is a paucity of data when it comes to populations at a higher baseline bleeding risk, such as patients with nonvalvular atrial fibrillation (NVAF) treated with direct oral anticoagulants (DOACs). This knowledge gap is important given the predominant role of DOACs for the prevention of stroke in NVAF^7^ and the common concomitant use of DOACs and NSAIDs.^8^ To address this knowledge gap, we conducted a large, multinational cohort study to assess the risk of GI bleeding associated with the concomitant use of DOACs and COX-2–selective NSAIDs compared with concomitant use of DOACs and nonselective NSAIDs among patients with NVAF.

## Methods

### Data Sources

This cohort study used data from the UK Clinical Practice Research Datalink (CPRD) Aurum and the Régie de l’assurance maladie du Québec between 2011 and 2020. The study protocol was approved by CPRD’s Independent Scientific Advisory Committee, Institut de la statistique du Québec, and the Research Ethics Board of the Jewish General Hospital. The requirement for informed consent was waived because the data were deidentified. We followed the Strengthening the Reporting of Observational Studies in Epidemiology (STROBE) reporting guideline for cohort studies.

The CPRD is the world’s largest primary care electronic medical record (EMR) database, including medical records of 60 million patients (18 million currently registered) seen across more than 2000 general practices. It is representative of the UK’s general population^9,10^ and contains all medication prescriptions issued by general practitioners, clinical measures (eg, blood pressure [BP]), laboratory findings, anthropometric measures (eg, body mass index [BMI, calculated as weight in kilograms divided by height in meters squared]), and lifestyle variables (eg, smoking).^9^ It also contains medical diagnoses coded by Systematized Nomenclature of Medicine–Clinical Terms, Read codes, and local EMIS web codes, all of which are more granular than the *International Classification of Diseases* system.^10,11^ The database is linked to the Hospital Episode Statistics, Office for National Statistics, and Index of Multiple Deprivation databases. The Hospital Episode Statistics database provides information about patient hospitalizations in the UK, including admissions, procedures, and discharge diagnoses (coded using *International Statistical Classification of Diseases, Tenth Revision* [*ICD-10*] codes). The Office for National Statistics database contains vital statistics data, including date, place, and underlying cause of death (coded using *ICD-10* codes) for all citizens living in the UK. The Index of Multiple Deprivation database contains information on socioeconomic status.

Régie de l’assurance maladie du Québec includes claims data of all residents in Quebec aged 65 years or older, as well as those without private insurance plans and recipients of financial assistance. It contains demographics, outpatient diagnoses (coded using the *International Classification of Diseases, Ninth Revision* and *ICD-10*), outpatient procedures, and outpatient dispensed drug prescriptions. The database is linked to the Maintenance et exploitation des données pour l’étude de la clientèle hospitalière, which contains information on hospital admissions, procedures, and discharge diagnoses (coded using the *ICD-10*), and the Institut de la statistique du Québec, which contains vital statistics data.^12^

### Study Cohort

Our source population included all patients diagnosed with AF between January 1, 2011 (when dabigatran was the first DOAC approved for stroke prevention in NVAF in the UK and Canada), and June 22, 2020 (latest date of data availability in the UK EMR database), or December 12, 2020 (latest date of data availability in Quebec claims database). We excluded patients younger than 18 years, diagnosed with valvular heart disease at any time before the AF diagnosis, or diagnosed with hyperthyroidism in the year before the AF diagnosis to restrict our population to adult patients with NVAF. We also excluded patients with a database history of less than 365 days to ensure sufficient time to assess exclusion criteria and covariates.

From this source population, we assembled a study cohort of all patients who initiated concomitant use of a DOAC (apixaban, dabigatran, edoxaban, rivaroxaban) and an NSAID. Concomitant use was defined as an overlap between a prescription for a DOAC and a prescription for an NSAID of at least 1 day. Cohort entry was defined as the first date of concomitant use. Patients could enter the cohort by either adding on an NSAID while using a DOAC, adding on a DOAC while using an NSAID, or initiating both drug classes simultaneously. The study design is illustrated in eFigures 1 and 2 in Supplement 1. Patient data were followed from cohort entry until the occurrence of an outcome, treatment discontinuation or switch, administrative censoring (eg, leaving the general practice in the UK EMR database), death, or the end of the study period, whichever occurred first. Due to the expected short duration of concomitant use, patients were allowed to contribute multiple episodes of concomitant use to the study, with patients able to reenter the cohort upon reinitiation of concomitant use at least 6 months after the last episode to minimize potential carryover effects.

### Exposure Definition

We used an as-treated exposure definition, where patients were considered continuously exposed to concomitant use if the prescription durations of both drugs of interest (DOACs and NSAIDs) were overlapping each other. Prescription duration was calculated based on the intended duration of use or defined daily doses. In the event of nonoverlap, we allowed a 30-day grace period between successive prescriptions. The exposure groups were (1) concomitant use of DOACs and COX-2–selective NSAIDs (celecoxib, diclofenac, etodolac, mefenamic acid, meloxicam, rofecoxib)^13^ and (2) concomitant use of DOACs and nonselective NSAIDs (fenoprofen, flurbiprofen, ibuprofen, indomethacin, ketoprofen, ketorolac, nabumetone, naproxen, oxaprozin, oxyphen-butazone, phenylbutazone, piroxicam, sulindac, tenoxicam, tolmetin, tiaprofenic acid).^13,14,15^ A treatment switch was defined by a prescription for a COX-2–selective NSAID in the nonselective NSAID group or vice versa. Patients were permitted to switch from 1 DOAC to another. Switches from DOACs to vitamin K antagonists were handled as DOAC discontinuation.

### Outcome Definition

The primary outcome was GI bleeding, defined as hospitalization for a GI bleed. The event date was the date of hospital admission. Given previous studies suggesting that NSAIDs may also be associated with non-GI bleeding,^16^ we included non-GI bleeding as a secondary outcome, defined as hospitalization for a non-GI bleed, and with the event date being the date of hospital admission. For both outcomes, we used *ICD-10* diagnostic codes in any position in the hospitalization record (eTable 1 in Supplement 1).

### Covariates

We adjusted for the following potential confounders measured at cohort entry: calendar year, time since NVAF diagnosis, age (modeled flexibly using restricted cubic splines), sex, alcohol-related disorders, hypertension, prior ischemic stroke or transient ischemic attack, congestive heart failure, coronary artery disease, peripheral vascular disease, prior major bleeding, type 2 diabetes, liver disease, and kidney disease (all comorbidities diagnosed at any time before cohort entry). Cancer (other than nonmelanoma skin cancer) was considered if diagnosed in the year before cohort entry. We further adjusted for use of antiplatelet agents, selective serotonin reuptake inhibitors, proton pump inhibitors, histamine-2 blockers, vitamin K antagonists, and systemic corticosteroids in the year before cohort entry. Finally, we adjusted for the number of hospitalizations in the year before cohort entry as a proxy for overall health status. In the UK EMR database, we additionally considered smoking (current, former, never, unknown), BMI (<25, 25-29, ≥30, unknown), and BP (systolic BP ≥130 mm Hg or diastolic BP ≥80 mm Hg, systolic BP <130 mm Hg and diastolic BP <80 mm Hg, unknown) using the last measurement before cohort entry, and Index of Multiple Deprivation quintiles as indicators of socioeconomic status. In the Quebec claims database, we additionally considered the number of nonanticoagulant drugs in the year before cohort entry. Information on race and ethnicity is not regularly captured in the 2 data sources and were therefore not considered in the analyses.

### Statistical Analysis

Data were analyzed between November 19, 2024, and July 25, 2025. Crude incidence rates (IRs) with 95% CIs for the outcomes were calculated for each exposure group, assuming a Poisson distribution. We used inverse probability of treatment weighting (IPTW) based on propensity scores to control for confounding. Propensity scores were calculated using multivariable logistic regression, estimating the probability of receiving DOACs and COX-2–selective NSAIDs vs DOACs and nonselective NSAIDs, conditional on all aforementioned covariates. Propensity scores were calculated separately for the different strata defined based on the type of cohort entry (DOAC users adding NSAIDs vs NSAID users adding DOACs or coinitiators of DOACs and NSAIDs). Extreme weights were trimmed at the 99th percentile. Covariate imbalance after IPTW was assessed using standardized mean differences; covariates with standardized mean differences of 0.1 or greater after IPTW were included in the outcome models. Cox proportional hazards models were used to estimate site-specific weighted hazard ratios (HRs) and 95% CIs of the study outcomes; the estimates were pooled together using random-effects models.^17^ Because patients were allowed to contribute multiple episodes of concomitant use, we calculated IPTW for every episode separately and used a robust sandwich estimator to estimate the variance due to correlated data. Analyses were conducted using SAS, version 9.4 (SAS Institute Inc). Findings were considered significant if the 95% CI did not include 1.

#### Secondary Analyses

Several secondary analyses were performed. First, we stratified by age (<75 vs ≥75 years) and sex. Second, we stratified by the order of drug initiation (DOAC users adding NSAIDs vs NSAID users adding DOACs or coinitiators of DOACs and NSAIDs). Third, we stratified by baseline bleeding risk using a modified version of the HAS-BLED (hypertension, abnormal kidney or liver function, stroke, bleeding, elderly, and drugs or excess alcohol use) score.^18^ Finally, we stratified by individual DOACs (feasible for rivaroxaban and apixaban).

#### Sensitivity Analyses

We also performed several sensitivity analyses. First, to assess potential exposure misclassification, we used a 15-day grace period between nonoverlapping successive prescriptions (both for DOACs and NSAIDs). Second, to assess potential outcome misclassification, we used a stricter outcome definition based on hospitalization codes in the primary position only. Third, we additionally included fatal events using the primary cause of death in vital statistics data (Office for National Statistics and Institut de la statistique du Québec) as part of the definition of the outcomes. Fourth, to eliminate residual confounding due to prior events, we excluded patients with a history of major bleeding. Fifth, we used multiple imputation for missing values for BMI and BP in the UK EMR database. Sixth, we allowed patients to contribute their first treatment episode only. Finally, we excluded patients with noninfective enteritis, colitis, or ulcer as these patients may have a low probability of receiving nonselective NSAIDs.

## Results

**Figure 1.**
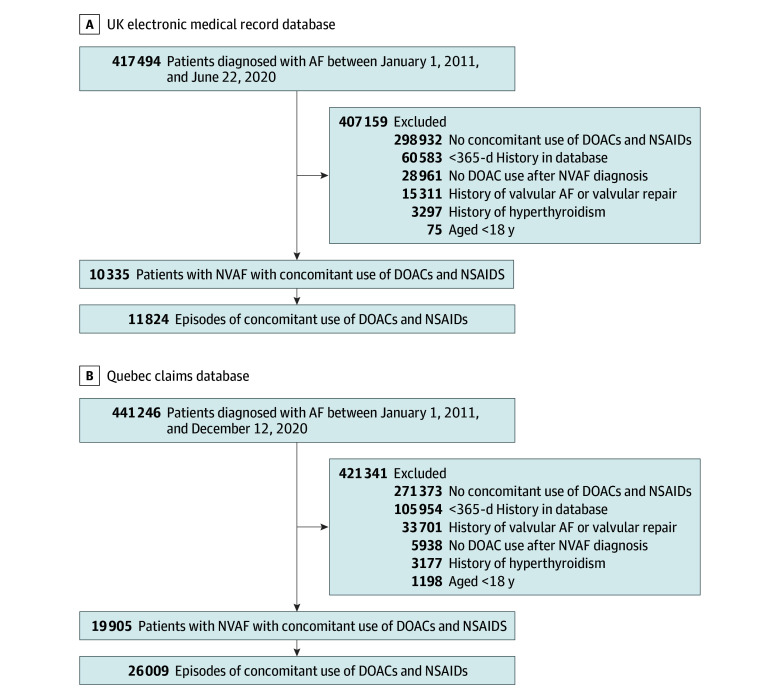
Flowcharts Illustrating the Construction of the Study Cohorts AF indicates atrial fibrillation; DOAC, direct oral anticoagulant; NSAID, nonsteroidal anti-inflammatory drug; NVAF, nonvalvular atrial fibrillation.

Our study cohort included 30 240 patients with NVAF who initiated concomitant use of DOACs and NSAIDs (10 335 in the UK and 19 905 in Quebec; mean [SD] age, 72.1 [9.2] years; 13 094 female [43.3%] and 17 146 male [56.7%]). They contributed 37 833 episodes of concomitant use of DOACs and NSAIDs, including 45.2% initiating DOACs and COX-2–selective NSAIDs and 54.8% initiating DOACs and nonselective NSAIDs (Figure 1). In the UK, diclofenac was the most common COX-2–selective NSAID (625 of 1566 episodes [39.9%]), and naproxen was the most common nonselective NSAID (6673 of 10 258 episodes [65.1%]). In Quebec, celecoxib was the most common COX-2–selective NSAID (13 302 of 15 539 episodes [85.6%]), and naproxen was the most common nonselective NSAID (8188 of 10 470 episodes [78.2%]) (eTable 2 in Supplement 1). Baseline characteristics were similar between exposure groups before IPTW and well balanced after IPTW (eTables 3 and 4 in Supplement 1).

Most patients (UK, 98.0%; Quebec, 93.8%) contributed up to 2 treatment episodes (eTable 5 in Supplement 1). Median follow-up was 44 days (IQR, 30-44 days) in the UK data and 30 days (IQR, 23-46 days) in the Quebec data. The Table shows that compared with concomitant use of DOACs and nonselective NSAIDs, concomitant use of DOACs and COX-2–selective NSAIDs was associated with a decreased risk of GI bleeding (unweighted IRs per 1000 person-years: UK, 20.47 [95% CI, 6.28-34.65] vs 34.77 [95% CI, 25.23-44.31]; Quebec, 27.04 [95% CI, 20.70-33.37] vs 52.52 [95% CI, 38.64-66.41]; pooled weighted HR, 0.63 [95% CI, 0.46-0.87]; *I*^2^ = 56%). Concomitant use of DOACs and COX-2–selective NSAIDs was further associated with a decreased risk of non-GI bleeding (unweighted IRs per 1000 person-years: UK, 17.92 [95% CI, 4.65-31.20] vs 34.82 [95% CI, 25.27-44.38]; Quebec, 31.31 [95% CI, 24.49-38.13] vs 66.92 [95% CI, 51.24-82.59]; pooled weighted HR, 0.54 [95% CI, 0.40-0.74]; *I*^2^ = 0%). The most common types of non-GI bleeding were hematuria (UK, 19 of 60 episodes [31.7%]; Quebec, 50 of 158 episodes [31.6%]) and bleeding as a complication of an intervention (UK, 9 of 60 episodes [15.0%]; Quebec, 50 of 158 episodes [22.2%]) (eTable 6 in Supplement 1).

**Table.** Risk of GI Bleeding and Non-GI Bleeding Associated With Concomitant Use of DOACs and COX-2–Selective NSAIDs vs Concomitant Use of DOACs and Nonselective NSAIDs Among Patients With NVAF

Variable	Episodes, No.	Events, No.	Patient-years	IR per 1000 person-years (95% CI)^a^	Crude HR (95% CI)	IPTW HR (95% CI)	Pooled weighted HR (95% CI)	*I*^2^, %
**GI bleeding**								
UK							0.63 (0.46-0.87)	56
DOACs plus COX-2–selective NSAIDs	1566	8	391	20.47 (6.28-34.65)	0.69 (0.32-1.48)	0.99 (0.51-1.95)		
DOACs plus nonselective NSAIDs	10 258	51	1467	34.77 (25.23-44.31)	1 [Reference]	1 [Reference]		
Quebec								
DOACs plus COX-2 selective NSAIDs	15 539	70	2589	27.04 (20.70-33.37)	0.58 (0.41-0.84)	0.56 (0.39-0.80)		
DOACs plus non-selective NSAIDs	10 470	55	1047	52.52 (38.64-66.41)	1 [Reference]	1 [Reference]		
**Non-GI bleeding**								
UK							0.54 (0.40-0.74)	0
DOACs plus COX-2–selective NSAIDs	1566	7	391	17.92 (4.65-31.20)	0.57 (0.26-1.28)	0.51 (0.21-1.24)		
DOACs plus nonselective NSAIDs	10 258	51	1465	34.82 (25.27-44.38)	1 [Reference]	1 [Reference]		
Quebec								
DOACs plus COX-2 selective NSAIDs	15 539	81	2587	31.31 (24.49-38.13)	0.54 (0.39-0.75)	0.55 (0.39-0.76)		
DOACs plus nonselective NSAIDs	10 470	70	1046	66.92 (51.24-82.59)	1 [Reference]	1 [Reference]		

Abbreviations: COX-2, cyclooxygenase 2; DOAC, direct oral anticoagulant; GI, gastrointestinal; HR, hazard ratio; IPTW, inverse probability of treatment weighting; IR, incidence rate; NSAID, nonsteroidal anti-inflammatory drug; NVAF, nonvalvular atrial fibrillation.

^a^ Calculated based on the unweighted cohort.

**Figure 2.**
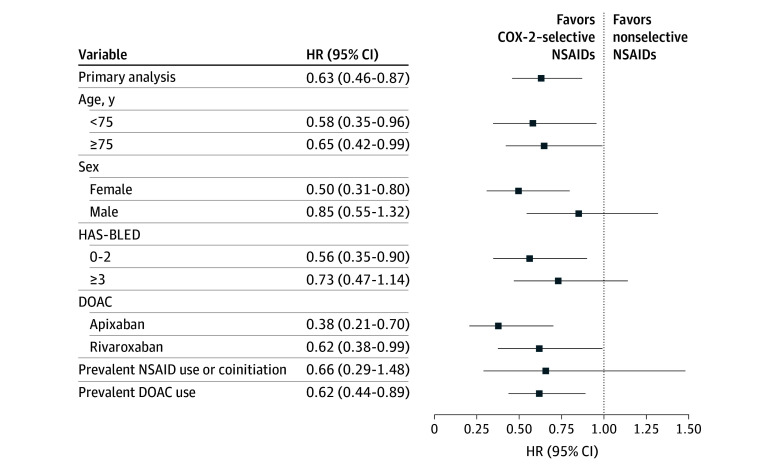
Forest Plot Summarizing the Secondary Analyses COX-2 indicates cyclooxygenase 2; DOAC, direct oral anticoagulant; HAS-BLED, hypertension, abnormal kidney or liver function, stroke, bleeding, elderly, and drugs or excess alcohol use; HR, hazard ratio; NSAID, nonsteroidal anti-inflammatory drug.

**Figure 3.**
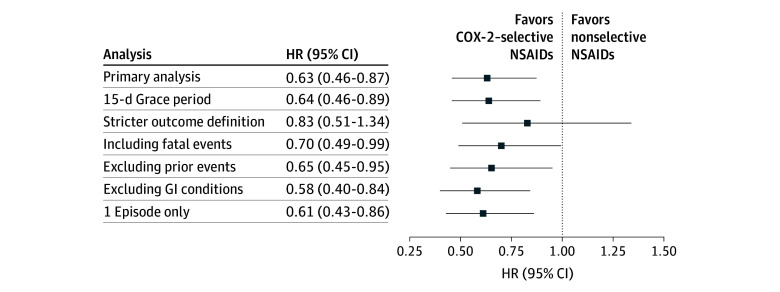
Forest Plot Summarizing Sensitivity Analyses COX-2 indicates cyclooxygenase 2; GI, gastrointestinal; HR, hazard ratio; NSAID, nonsteroidal anti-inflammatory drug.

There was no major effect modification by age, order of drug initiation in concomitant use, baseline bleeding risk, or individual DOACs (eTables 7 and 8 in Supplement 1; Figure 2). However, female patients experienced a significant decreased risk of GI bleeding associated with concomitant use of DOACs and COX-2–selective NSAIDs (pooled weighted HR, 0.50 [95% CI, 0.31-0.80]; *I*^2^ = 0%) compared with male patients (pooled weighted HR, 0.85 [95% CI, 0.55-1.32]; *I*^2^ = 78%) (eTable 7 in Supplement 1; Figure 2). The results of the sensitivity analyses were overall consistent with the primary analysis, with individual pooled weighted HRs ranging from 0.58 (95% CI, 0.40-0.84) when excluding prior GI conditions and 0.83 (95% CI, 0.51-1.34) when using a stricter outcome definition (eTable 9 in Supplement 1; Figure 3).

## Discussion

This multinational cohort study found an overall 37% decrease in the risk of GI bleeding associated with the concomitant use of DOACs and COX-2–selective NSAIDs vs concomitant use of DOACs and nonselective NSAIDs among patients with NVAF. Moreover, the decreased GI bleeding risk with DOACs and COX-2–selective NSAIDs seemed to be more pronounced among female patients. Sensitivity analyses yielded findings that were overall consistent with the primary analyses. Furthermore, a decreased risk with DOACs and COX-2–selective NSAIDs was also associated with non-GI bleeding as a secondary outcome.

Our findings suggest that the beneficial effects of COX-2–selective NSAIDs vs nonselective NSAIDs regarding the risk of GI bleeding that were first shown in randomized clinical trials among patients with indications for analgesic treatment^4,5,6^ are retained among patients with NVAF who are concomitantly treated with DOACs. Of note, the magnitude of the effect seems to be retained to a large extent as well (37% risk reduction in our study vs 29%-60% risk reductions in the trials^4,5,6^). This finding is important given the high baseline risk of GI bleeding during treatment with DOACs. At the pharmacologic level, our findings are in accordance with the important role of COX-1 for the homeostasis of the GI mucosa both in physiologic and pathophysiologic conditions,^19^ suggesting that selective inhibition of COX-2 may lead to fewer GI toxic effects and less bleeding, even in settings of ongoing DOAC-induced inhibition of the coagulation cascade.^20^ Fewer GI toxic effects and less bleeding are relevant given the scarcity of therapeutic strategies (eg, proton pump inhibitors) for the mitigation of DOAC-related GI bleeding.^21^

Of note, the decrease in the risk of GI bleeding associated with concomitant use of DOACs and COX-2–selective NSAIDs was more pronounced among female patients, albeit with variation between databases. While this observation requires replication and should be considered as hypothesis generating, it may be associated with potential differences in the risk of DOAC-induced GI bleeding between sexes. Indeed, a higher risk of DOAC-induced GI bleeding among women has been suggested,^22^ which would make COX-2 selectivity in NSAIDs and the resulting alleviation of GI toxic effects even more relevant in this setting. However, studies assessing sex-specific effects of DOACs have yielded overall inconsistent findings.^22,23^ Moreover, the aforementioned variation between databases suggests that differences in regional clinical practices or other factors may have also contributed to the observed treatment effect heterogeneity.

Our results show that COX-2–selective NSAIDs may be beneficial among DOAC users in reducing the risk of non-GI bleeding. While robust mechanistic evidence is lacking, following pharmacologic considerations could be relevant. First, platelet dysfunction due to COX-1 inhibition has been described for NSAIDs such as ibuprofen, albeit alleviated when compared with genuine antiplatelet agents such as low-dose acetylsalicylic acid.^24^ Platelet dysfunction could then lead to increases in the risk of bleeding regardless of the organ system. Second, while COX-1 is expressed in most cells in the organism, COX-2 shows a more limited expression pattern.^25^ Hence, disruptions of COX-1–related pathways may be more likely to affect different organ systems.

Two post hoc analyses of randomized clinical trials assessed the risk of GI bleeding with concomitant use of DOACs and NSAIDs vs concomitant use of vitamin K antagonists and NSAIDs among patients with NVAF, showing no differences between groups.^26,27^ The results of these analyses are important for clinical decision-making as they suggest that concomitant use of NSAIDs may not affect the comparative safety between DOACs and vitamin K antagonists with respect to the risk of GI bleeding. However, they do not answer the question about the potential benefits of COX-2 selectivity among DOAC users requiring treatment with NSAIDs. Such benefits are particularly important given the predominant role of DOACs for stroke prevention in NVAF.^28^

### Strengths and Limitations

Our study had several strengths. First, it is the first to our knowledge to investigate the role of COX-2 selectivity in NSAIDs and the risk of GI bleeding among patients with NVAF treated with DOACs. Thus, our study sheds light on a clinically important question. Second, the use of 2 databases from 2 different countries and health care systems provided us with a sufficient sample size and allowed us to estimate relatively precise effect estimates with high external validity. That being said, it should be noted that the pooled estimates for the primary outcome were mostly driven by the findings from Quebec. Moreover, analyses focusing on specific COX-selective compounds were not feasible. Third, we used a common study protocol in both databases, which enabled us to pool together site-specific findings.

Our study also had several limitations. First, the assessment of medication use was based on prescription or dispensing data from electronic health care databases and not actual intake, which may have led to exposure misclassification. However, a sensitivity analysis with an alternate grace period showed consistent findings. Second, outcome misclassification is possible. To assess the potential impact of this bias, we performed sensitivity analyses that applied stricter or expanded outcome definitions. Reassuringly, the respective findings were comparable to those in the primary analysis. Third, residual confounding due to unmeasured confounders, such as frailty, or not routinely available confounders, such as dose, could not be ruled out despite our best efforts to control for this bias using an active comparator and propensity score–based IPTW.

## Conclusions

This cohort study found that in patients with NVAF treated with DOACs, COX-2–selective NSAIDs seemed to retain their benefits over nonselective NSAIDs in reducing the risk of GI bleeding. Future studies should replicate our findings, as well as complement them with analyses regarding the impact of COX-2 selectivity on the risk of stroke among patients treated with DOACs.

## Notes

Supplement 1. **eFigure 1.** Study Design **eFigure 2.** Study Cohort Entry **eTable 1.** *ICD-9* and *ICD-10* Codes for Inclusion and Exclusion Criteria, Outcomes, and Comorbidities **eTable 2.** Distribution of NSAIDs in the Study Cohort **eTable 3.** Baseline Characteristics of Patients in the UK Electronic Medical Record Database **eTable 4.** Baseline Characteristics of Patients in the Quebec Claims Database **eTable 5.** Treatment Episodes per Patient **eTable 6.** Distribution of Types of Nongastrointestinal Bleeding in the Study Cohort **eTable 7.** Risk of GI Bleeding Associated With Concomitant Use of DOACs and COX-2–Selective NSAIDs vs Concomitant use of DOACs and Nonselective NSAIDs Among Patients With NVAF (Stratification by Demographics) **eTable 8.** Risk of GI Bleeding With Concomitant Use of DOACs and COX-2–Selective NSAIDs vs Concomitant use of DOACs and Nonselective NSAIDs Among Patients With NVAF (Stratification by User Type, Baseline Bleeding Risk, and Individual DOACs) **eTable 9.** Risk of GI Bleeding With Concomitant Use of DOACs and COX-2–Selective NSAIDs vs Concomitant use of DOACs and Nonselective NSAIDs Among Patients With NVAF (Sensitivity Analyses)

Supplement 2.
Data Sharing Statement
