# Supplementary material for: Direct Oral Anticoagulants, COX-2–Selective NSAIDs, and Gastrointestinal Bleeding in Atrial Fibrillation
Source: JAMA Netw Open. 2026 May 26;9(5):e2613941. doi: 10.1001/jamanetworkopen.2026.13941 (PMC13213523; doi:10.1001/jamanetworkopen.2026.13941)
Supplement: Supplement 1. — eFigure 1. Study Design eFigure 2. Study Cohort Entry eTable 1. ICD-9 and ICD-10 Codes for Inclusion and Exclusion Criteria, Outcomes, and Comorbidities eTable 2. Distribution of NSAIDs in the Study Cohort eTable 3. Baseline Characteristics of Patients in the UK Electronic Medical Record Database eTable 4. Baseline Characteristics of Patients in the Quebec Claims Database eTable 5. Treatment Episodes per Patient eTable 6. Distribution of Types of Nongastrointestinal Bleeding in the Study Cohort eTable 7. Risk of GI Bleeding Associated With Concomitant Use of DOACs and COX-2–Selective NSAIDs vs Concomitant use of DOACs and Nonselective NSAIDs Among Patients With NVAF (Stratification by Demographics) eTable 8. Risk of GI Bleeding With Concomitant Use of DOACs and COX-2–Selective NSAIDs vs Concomitant use of DOACs and Nonselective NSAIDs Among Patients With NVAF (Stratification by User Type, Baseline Bleeding Risk, and Individual DOACs) eTable 9. Risk of GI Bleeding With Concomitant Use of DOACs and COX-2–Selective NSAIDs vs Concomitant use of DOACs and Nonselective NSAIDs Among Patients With NVAF (Sensitivity Analyses) [file jamanetwopen-e2613941-s001.pdf]

Supplemental Online Content

Meinert FM, Dimakos J, Cui Y, Filion KB, Renoux C, Douros A. Direct oral anticoagulants, COX-2–selective NSAIDs, and gastrointestinal bleedin in atrial fibrillation. *JAMA Netw Open*. 2026;9(5):e2613941. doi:10.1001/jamanetworkopen.2026.13941

eFigure 1. Study Design

eFigure 2. Study Cohort Entry

eTable 1. *ICD-9* and *ICD-10* Codes for Inclusion and Exclusion Criteria, Outcomes, and Comorbidities

eTable 2. Distribution of NSAIDs in the Study Cohort

eTable 3. Baseline Characteristics of Patients in the UK Electronic Medical Record Database

eTable 4. Baseline Characteristics of Patients in the Quebec Claims Database

eTable 5. Treatment Episodes per Patient

eTable 6. Distribution of Types of Nongastrointestinal Bleeding in the Study Cohort

eTable 7. Risk of GI Bleeding Associated With Concomitant Use of DOACs and COX-2–Selective NSAIDs vs Concomitant use of DOACs and Nonselective NSAIDs Among Patients With NVAF (Stratification by Demographics)

eTable 8. Risk of GI Bleeding With Concomitant Use of DOACs and COX-2–Selective NSAIDs vs Concomitant use of DOACs and Nonselective NSAIDs Among Patients With NVAF (Stratification by User Type, Baseline Bleeding Risk, and Individual DOACs)

eTable 9. Risk of GI Bleeding With Concomitant Use of DOACs and COX-2–Selective NSAIDs vs Concomitant use of DOACs and Nonselective NSAIDs Among Patients With NVAF (Sensitivity Analyses)

This supplemental material has been provided by the authors to give readers additional information about their work.

© 2026 Meinert FM et al. *JAMA Network Open*.

eFigure 1. Study Design

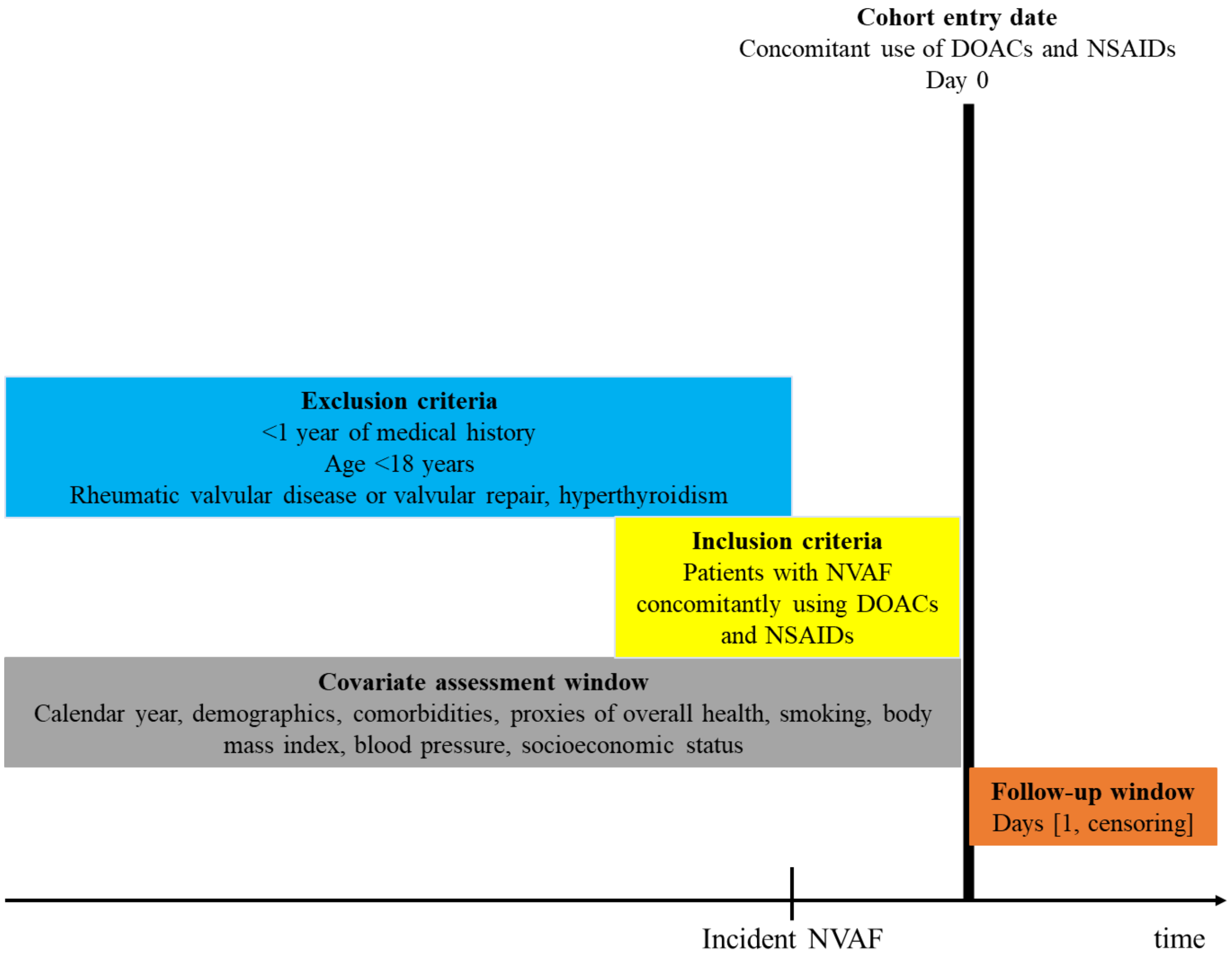

Abbreviations: DOACs, direct oral anticoagulants; NSAIDs, non steroidal anti-inflammatory drugs; NVAF, non-valvular atrial fibrillation.

© 2026 Meinert FM et al. *JAMA Network Open*.

eFigure 2. Study Cohort Entry

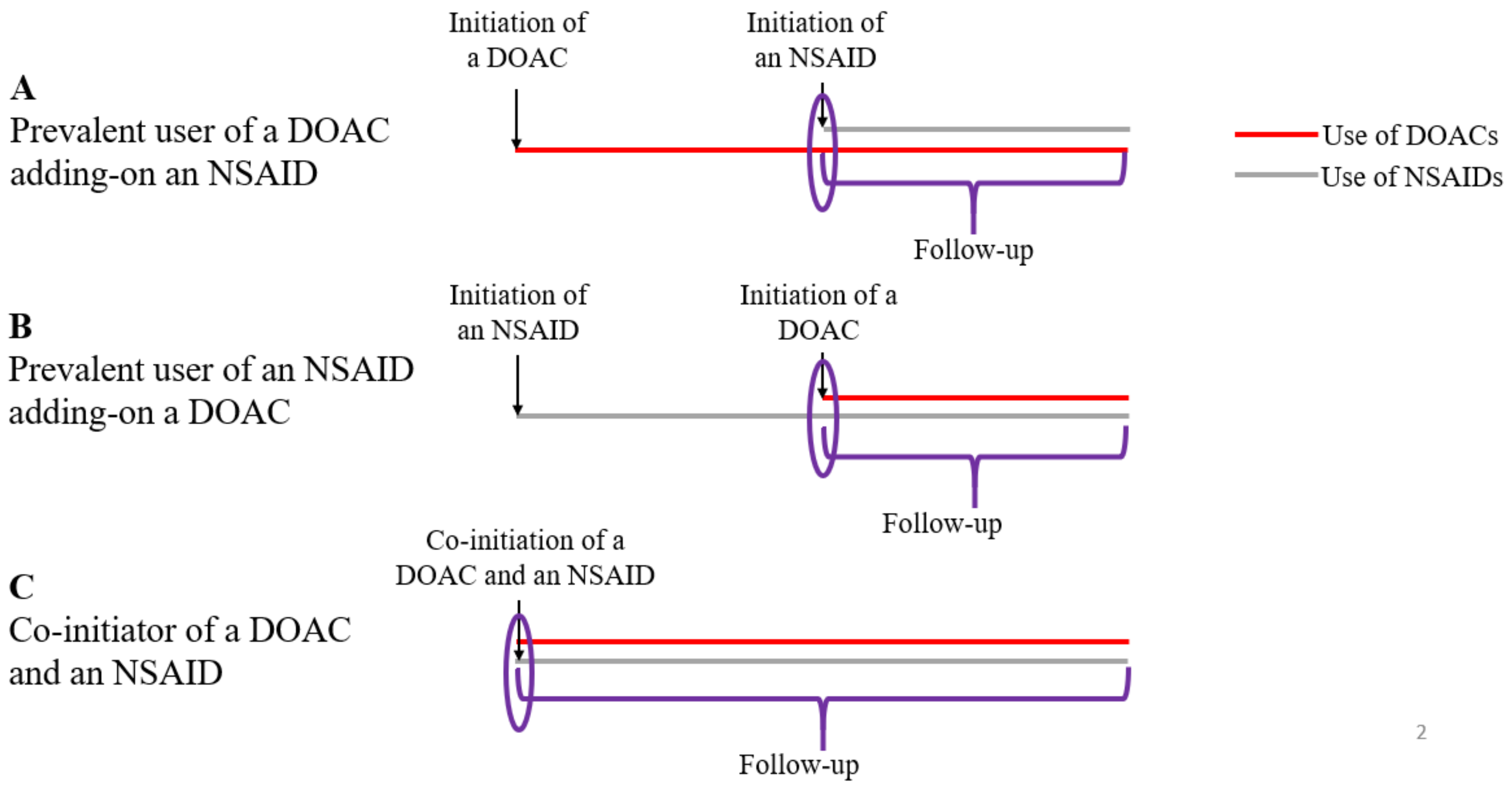

Patient A. Patient is treated with a DOAC after the diagnosis of NVAF and then adds-on an NSAID, initiating concomitant use of the two drug classes for the first time and entering the study cohort.
Patient B. Patient is treated with an NSAID after the diagnosis of NVAF and then adds-on a DOAC, initiating concomitant use of the two drug classes for the first time and entering the study cohort.
Patient C. Patient co-initiates a DOAC and an NSAID on the same day after the diagnosis of NVAF for the first time, entering the study cohort.

Abbreviations: DOACs, direct oral anticoagulants; NSAIDs, non steroidal anti-inflammatory drugs; NVAF, non-valvular atrial fibrillation.

© 2026 Meinert FM et al. *JAMA Network Open*.

eTable 1. ***ICD-9*** **and** ***ICD-10*** **Codes for Inclusion and Exclusion Criteria, Outcomes, and Comorbidities**

Variable type	*ICD-9* codes[3]	*ICD-10* codes[3]
**Inclusion criteria**[1]		
Atrial fibrillation and flutter	427.3, 427.31, 427.32	148
**Exclusion criteria**[1]		
Valvular mitral or aortic heart disease, valvular repair, and procedures	349, 395, 396, 424.0, 424.1, V43.3, V42.2, 35.0, 35.1, 35.2, 746.3, 746.4, 746.5, 746.6	I05, I06, I34, I35, I08.0, Z95.2, Z95.3, Q23
Hyperthyroidism[2]	242	E05
**Outcomes**[2]		
Gastrointestinal bleeding	Not applicable	I850, K920, K921, K922, K2211, K226, K228, K250, K252, K254, K256, K260, K262, K264, K266, K270, K272, K274, K276, K280, K282, K284, K286, K290, K294, K2921, K2961, K2971, K2991, K2981, K3181, K5711, K5713, K5731, K5733, K661, K625, K552
Non-gastrointestinal bleeding	Not applicable	D699, H0289, H0523, H113, H313, H356, H431, H44819, I60, I61, I621, I620, I629, I230, I312, J94.2, M250, M7981, N02, N3289, N92, N950, N837, N939, R040, R041, R042, R048, R049, R233, R31, R58, T792, T810
**Comorbidities**[1]		
Alcohol-related disorders	291, 303, 357.5, 425.5, 535.3, 571.0, 571.1, 571.2, 571.3, 790.3, 977.3, 980.0, 980.9, V79.1	E24.4, F10.1, F10.2, F10.3, F10.4, F10.5, F10.6, F10.7, F10.8, F10.9, G31.2, G62.1, G72.1, I42.6, K29.2, K70, K85.2, K86.0, O35.4, R78.0, T51.0, T51.9, X65, Y57.3, Y90, Y91, Z50.2, Z71.4, Z72.1
Cancer (excl non-melanoma skin cancer)	174-209	C45-C97
Congestive heart failure	428x, 402.01, 402.11, 402.91, 404.01, 404.03, 404.11, 404.13, 404.91, 404.93	I50, I11.0, I13.0, I13.2
Coronary artery disease	410, 411, 412, 413, 414	I20, I21, I22, I24, I25
Diabetes mellitus	250x	E10x-E14x

© 2026 Meinert FM et al. *JAMA Network Open*.

Variable type	*ICD-9* codes[3]	*ICD-10* codes[3]
Hyperlipidemia	272.0-272.4	E78.0-E78.5
Hypertension	401x-404x	I10x-I13x
Liver disease	006.3, 070, 070.0, 070.20, 070.21, 070.22, 070.23, 070.32, 070.33, 070.41, 070.42, 070.43, 070.44, 070.49, 070.54, 070.6, 070.71, 072.71, 091.62, 122.0, 122.5, 122.8, 130.5, 155, 197.7, 275.01, 275.02, 275.03, 453.0, 456.0, 456.1, 456.2 , 570, 571.0, 571.1, 571.2, 571.3, 571.4, 571.5, 571.6, 571.8, 571.9, 572, 572.2, 572.3, 572.4, 572.8, 573, 573.0, 573.4, 573.5, 789.1 , 790.4, 996.82, V42.7	A06.4, A51.45, B00.81, B15.0, B16.0, B16.2, B17.11, B18, B19.0, B19.11, B19.21, B15-B19, B25.1, B26.81, B58.1, B67.0, B67.5, B67.8, B94.2, C22.0, C22.1, C22.3, C22.4, C22.7, C22.9, C78.7, E83.0, E83.1, I82.0, I85, I86.4, K70, K70.0, K70.1, K70.2, K70.3, K70.4, K70.9, K71, K71.1, K71.3, K71.4, K71.5, K71.7, K72, K73, K74.0, K74.1, K74.2, K74.3, K74.4, K74.5, K74.6, K75, K75.8, K76, K76.0, K76.1, K76.2, K76.3, K76.4, K76.5, K76.6, K76.7, K76.81, K77, R16.0, R16.2, R74.0, T86.4, Z94.4
Peripheral arterial disease	440, 441, 445	I70, I71
Prior ischemic stroke/TIA	433.01, 433.11, 433.21, 433.31, 433.81, 433.91, 434.01, 434.11, 434.91, 362.31, 362.32, 435, 362.34, V12.54	I63, I64, H34.1
Renal disease	585, 586	N18, N19

Abbreviations: *ICD-9*, *International Classification of Diseases, Ninth Revision*; *ICD-10*, *International Statistical Classification of Diseases, Tenth Revision*.

[1] The definition of inclusion criteria, exclusion criteria, and comorbidities was based on inpatient and outpatient diagnoses. For hyperthyroidism, diabetes mellitus, and hyperlipidemia, use of specific medications (antithyroid agents, antidiabetic drugs, antihyperlipidemic drugs) was additionally considered. In the UK data source, medication use was documented via Product codes (not shown). In the Quebec data source, medication use was documented via DIN codes (not shown).
[2] The definition of the outcomes was based on inpatient diagnoses only.
[3] In the Quebec data source, outpatient diagnoses were documented via *ICD-9* and *ICD-10* codes. In the UK data source, outpatient diagnoses were documented via SNOMED Clinical terms, Read codes, and local EMIS Read codes (not shown). In both data sources, inpatient diagnoses were documented via *ICD-10* codes.

© 2026 Meinert FM et al. *JAMA Network Open*.

eTable 2. Distribution of NSAIDs in the Study Cohort

Compound	UK n (%)	Quebec n (%)
COX-2 selective NSAIDs		
Celecoxib	210 (13)	13,302 (86)
Diclofenac	625 (40)	1,938 (12)
Meloxicam	396 (25)	294 (2)
Other	335 (22)	5 (1)
Non-selective NSAIDs		
Ibuprofen	2,520 (25)	1,448 (14)
Naproxen	6,673 (65)	8,188 (78)
Other	1,052 (10)	834 (8)
Multiple compounds	13 (1)	0
Total number	11,824	26,009

Abbreviations: NSAIDs, non steroidal anti-inflammatory drugs..

© 2026 Meinert FM et al. *JAMA Network Open*.

eTable 3. Baseline Characteristics of Patients in the UK Electronic Medical Record Database

	Before IPTW DOACs + COX-2 selective NSAIDs (n=1,566)	DOACs + non selective NSAIDs (n=10,258)	SMD	After IPTW DOACs + COX-2 selective NSAIDs (n=1,563)	DOACs + non selective NSAIDs (n=10,259)	SMD
Age in years, mean (standard deviation)	71.67 (10.5)	71.39 (10.1)	0.027	71.44 (10)	71.42 (10.1)	0.002
Female sex	689 (44)	3,898 (38)	0.122	613 (39.3)	3,980 (38.8)	0.009
Calendar year of cohort entry						
2011-2016	758 (48.4)	4,604 (44.9)	0.071	693 (44.3)	4,650 (45.3)	0.020
2017-2019	808 (51.6)	5,654 (55.1)	-0.071	870 (55.7)	5,609 (54.7)	0.020
Smoking status						
Current	S	S	-0.027	S	S	-0.009
Former	652 (41.6)	4,444 (43.3)	-0.034	672 (43)	4,422 (43.1)	-0.002
Never	811 (51.8)	5,070 (49.4)	0.047	783 (50.1)	5,102 (49.7)	0.007
Unknown	S	S	0.004	S	S	-0.005
Body mass index in kg/m^2						
<25	288 (18.4)	1.963 (19.1)	-0.019	302 (19.3)	1,953 (19.1)	0.007
25-29	511 (32.6)	3.644 (35.5)	-0.061	537 (34.3)	3,603 (35.1)	-0.016
≥30	732 (46.7)	4,429 (43.2)	-0.072	691 (44.2)	4,480 (43.7)	0.011
Unknown	35 (2.2)	222 (2.2)	0.005	33 (2.1)	223 (2.2)	-0.003
Blood pressure control*						
Normal	430 (27.5)	S	-0.068	461 (29.5)	S	-0.014
High	1.136 (72.5)	7.125 (69.5)	0.068	1.102 (70.5)	7.168 (69.9)	0.014
Unknown	0 (0)	S	-0.014	0	S	-0.014
**Comorbidities**						
Alcohol-related disorders	455 (29.1)	3.335 (32.5)	-0.075	495 (31.7)	3.289 (32.1)	-0.008
Arterial hypertension	1.163 (74.3)	7.789 (75.9)	-0.038	1.182 (75.6)	7.766 (75.7)	-0.001
Ischemic stroke/TIA**	273 (17.4)	2.075 (20.2)	-0.072	304 (19.5)	2.036 (19.9)	0.010
Congestive heart failure	341 (21.8)	2.563 (25)	-0.076	373 (23.9)	2.518 (24.6)	-0.016
Coronary artery disease	478 (30.5)	3.608 (35.2)	-0.099	539 (34.5)	3.545 (34.6)	-0.002
Peripheral vascular disease	107 (6.8)	934 (9.1)	-0.084	134 (8.6)	903 (8.8)	-0.007

© 2026 Meinert FM et al. *JAMA Network Open*.

	Before IPTW DOACs + COX-2 selective NSAIDs (n=1,566)	DOACs + non selective NSAIDs (n=10,258)	SMD	After IPTW DOACs + COX-2 selective NSAIDs (n=1,563)	DOACs + non selective NSAIDs (n=10,259)	SMD
Major bleeding	62 (4)	316 (3.1)	0.048	52 (3.3)	329 (3.2)	0.007
Diabetes mellitus	446 (28.5)	3.047 (29.7)	-0.027	467 (29.9)	3.033 (29.6)	0.007
Liver disease	66 (4.2)	529 (5.2)	-0.045	78 (5)	516 (5)	-0.003
Renal disease	805 (51.4)	4,986 (48.6)	0.056	765 (49)	5.024 (49)	<0.001
Cancer	84 (5.4)	638 (6.2)	-0.037	98 (6.3)	628 (6.1)	0.007
**Comedications**						
Antiplatelet agents	435 (27.8)	2.712 (26.4)	0.030	413 (26.4)	2.731 (26.6)	-0.005
Selective serotonin reuptake inhibitors	180 (11.5)	1.026 (10)	0.048	157 (10.1)	1,046 (10.2)	-0.005
Proton pump inhibitors	1.027 (65.6)	5.869 (57.2)	0.173	916 (58.6)	5.984 (58.3)	0.006
H_2 blockers	101 (6.5)	504 (4.9)	0.067	83 (5.3)	525 (5.1)	0.008
Vitamin K antagonists	176 (11.2)	998 (9.7)	0.049	150 (9.6)	1.018 (9.9)	-0.011
Systemic corticosteroids	252 (16.1)	1,605 (15.7)	0.012	243 (15.6)	1.611 (15.7)	-0.004
N Hospitalizations in the past year						
0	1,157 (73.9)	7,793 (76)	-0.048	1,185 (75.8)	7,765 (75.7)	0.003
1	784 (18.1)	1,596 (15.6)	0.069	246 (15.7)	1,630 (15.9)	-0.004
≥2	125 (8)	869 (8.5)	-0.018	132 (8.5)	864 (8.4)	0.001
Index of multiple deprivation						
1	236 (15.1)	1.434 (14)	0.031	219 (14)	1.450 (14.1)	-0.003
2	265 (16.9)	1.443 (14)	0.079	235 (15)	1.485 (14.5)	0.015
3	205 (13.1)	1.517 (14.8)	-0.049	234 (15)	1.493 (14.6)	0.012
4	227 (14.5)	1.502 (14.6)	-0.004	223 (14.3)	1.498 (14.6)	-0.009
5	147 (9.4)	1.282 (12.5)	-0.100	182 (11.6)	1.239 (12.1)	-0.014
Unknown	486 (31)	3,080 (30)	0.022	470 (30.1)	3,094 (30.2)	-0.002
Time since NVAF diagnosis in months						
0-19	878 (56.1)	5.087 (49.6)	0.130	788 (50.4)	5.176 (50.5)	-0.001
≥20	688 (43.9)	5.171 (50.4)	-0.130	775 (49.6)	5.083 (49.5)	0.001
**Order of initiation of concomitant use**						
DOAC users adding-on an NSAID	1,016 (64.9)	7,911 (77.1)				

© 2026 Meinert FM et al. *JAMA Network Open*.

	Before IPTW<br>DOACs + COX-2 selective NSAIDs (n=1,566)	DOACs + non selective NSAIDs (n=10,258)	SMD	After IPTW<br>DOACs + COX-2 selective NSAIDs (n=1,563)	DOACs + non selective NSAIDs (n=10,259)	SMD
NSAID users adding-on a DOAC	448 (28.6)	1.875 (18.3)				
Co-initiation of DOACs and NSAIDs	102 (6.5)	472 (4.6)				

All values are n (%) unless indicated otherwise.
S = Data suppressed due to small cells.

* Normal blood pressure control was defined as systolic blood pressure <130 mmHg and diastolic blood pressure <80 mmHg, while high blood pressure was defined as systolic blood pressure ≥130 mmHg or diastolic blood pressure ≥80 mmHg.
** Mean (standard deviation) CHA2DS2-VASc score
Before IPTW: DOACs + COX-2 selective NSAIDs, 3.55 (1.75) vs DOACs + non selective NSAIDs, 3.64 (1.78)
After IPTW: DOACs + COX-2 selective NSAIDs, 3.62 (1.79) vs DOACs + non selective NSAIDs, 3.63 (1.78)

Abbreviations: IPTW, inverse-probability-treatment-weighting; SMD, standardized mean difference; DOACs, direct oral anticoagulants; COX-2, cyclooxygenase 2; NSAID, non steroidal anti-inflammatory drug; TIA, transient ischemic attack; NVAF, non-valvular atrial fibrillation; CHA2DS2-VASc, congestive heart failure, hypertension, age ≥75 years, diabetes mellitus, stroke, vascular disease, age 65-74 years, sex.

© 2026 Meinert FM et al. *JAMA Network Open*.

eTable 4. Baseline Characteristics of Patients in the Quebec Claims Database

	Before IPTW DOACs + COX-2 selective NSAIDs (n=15,539)	DOACs + non selective NSAIDs (n=10,470)	SMD	After IPTW DOACs + COX-2 selective NSAIDs (n=15,547)	DOACs + non selective NSAIDs (n=10,468)	SMD
Age in years, mean (standard deviation)	73.59 (8.3)	70.50 (9.3)	0.351	72.32 (8.9)	72.34 (8.9)	-0.002
Female sex (%)	7,677 (49.4)	4,110 (39.3)	0.205	7,027 (45.2)	4,728 (45.2)	0.001
Calendar year of cohort entry						
2011-2016	7,305 (47)	4,481 (42.8)	0.085	7.039 (45.3)	4,733 (45.2)	0.001
2017-2020	8,234 (53)	5.989 (57.2)	-0.085	8.508 (54.7)	5,735 (54.8)	-0.001
**Comorbidities**						
Alcohol-related disorders	653 (4.2)	598 (5.7)	-0.070	747 (4.8)	497 (4.8)	0.002
Arterial hypertension	14,839 (95.5)	9,933 (94.9)	0.029	14,808 (95.3)	9,975 (95.3)	-0.002
Ischemic stroke/TIA*	1,418 (9.2)	983 (9.4)	-0.009	1.442 (9.3)	976 (9.3)	-0.002
Congestive heart failure	3,737 (24.1)	2,734 (26.1)	-0.048	3,885 (25)	2,628 (25.1)	-0.003
Coronary artery disease	7,772 (50)	5,662 (54.1)	-0.081	8,048 (51.8)	5,433 (51.9)	-0.003
Peripheral vascular disease	1,869 (12)	1,299 (12.4)	-0.012	1,902 (12.2)	1,285 (12.3)	-0.001
Major bleeding	1,789 (11.5)	1,348 (12.9)	-0.042	1,873 (12.1)	1,265 (12.1)	-0.001
Diabetes mellitus	5,037 (32.4)	3,578 (34.2)	-0.037	5,170 (33.1)	3,495 (33.4)	-0.003
Liver disease	873 (5.6)	678 (6.5)	-0.036	936 (6)	632 (6)	-0.001
Renal disease	1,841 (11.9)	1,331 (12.7)	-0.026	1,906 (12.3)	1,289 (12.3)	-0.002
Cancer	2,708 (17.4)	2,084 (19.9)	0.063	2,871 (18.5)	1,935 (18.5)	-0.001
**Comedications**						
Antiplatelet agents	4,095 (26.4)	2,725 (26)	0.007	4,086 (26.3)	2,755 (26.3)	-0.001
Selective serotonin reuptake inhibitors	1,891 (12.2)	1,123 (10.7)	0.045	1,805 (11,6)	1,207 (11.5)	0.003
Proton pump inhibitors	9,388 (60.4)	5,998 (57.3)	0.064	9,208 (59.2)	6,214 (59.4)	-0.003
H_2 blockers	402 (2.6)	302 (2.9)	-0.018	422 (2.7)	286 (2.7)	-0.001
Vitamin K antagonists	1,795 (11.6)	945 (9)	0.083	1,643 (10.6)	1,115 (10.7)	-0.003
Systemic corticosteroids	3,219 (20.7)	2,112 (20.2)	0.014	3,204 (20.6)	2,173 (20.8)	-0.004
N Hospitalizations in the past year						
0	9,787 (63)	6,550 (62.6)	0.009	9,752 (62.7)	6.552 (62.6)	0.003

© 2026 Meinert FM et al. *JAMA Network Open*.

	**Before IPTW DOACs + COX-2 selective NSAIDs (n=15,539)**	**DOACs + non selective NSAIDs (n=10,470)**	**SMD**	**After IPTW DOACs + COX-2 selective NSAIDs (n=15,547)**	**DOACs + non selective NSAIDs (n=10,468)**	**SMD**
1	3,918 (25.2)	2,528 (24.2)	0.025	3,865 (24.9)	2,600 (24.8)	<0.001
≥2	1,834 (11.8)	1,392 (13.3)	-0.045	1,930 (12.4)	1,316 (12.6)	-0.005
Time since NVAF diagnosis in months						
0-25	7,822 (50.3)	5,072 (48.4)	0.038	7,691 (49.5)	5,151 (49.2)	0.005
≥26	7,717 (49.7)	5,398 (51.6)	-0.038	7,856 (50.5)	5,317 (50.8)	-0.005
**Order of initiation of concomitant use**						
DOAC users adding-on an NSAID	13,325 (85.8)	9,359 (89.4)				
NSAID users adding-on a DOAC	1,375 (8.9)	618 (5.9)				
Co-initiation of DOACs and NSAIDs	839 (5.4)	493 (4.7)				

All values are n (%) unless indicated otherwise.

* ** Mean (standard deviation) CHA2DS2-VASc score
Before IPTW: DOACs + COX-2 selective NSAIDs, 4.07 (1.44) vs DOACs + non selective NSAIDs, 3.83 (1.49)
After IPTW: DOACs + COX-2 selective NSAIDs, 3.97 (1.46) vs DOACs + non selective NSAIDs, 3.98 (1.48)

Abbreviations: IPTW, inverse-probability-treatment-weighting; SMD, standardized mean difference; DOACs, direct oral anticoagulants; COX-2, cyclooxygenase 2; NSAID, non steroidal anti-inflammatory drug; NVAF, non-valvular atrial fibrillation; TIA, transient ischemic attack; CHA2DS2-VASc, congestive heart failure, hypertension, age ≥75 years, diabetes mellitus, stroke, vascular disease, age 65-74 years, sex.

© 2026 Meinert FM et al. *JAMA Network Open*.

eTable 5. Treatment Episodes per Patient

Treatment episodes per patient	UK<br>n (%)	Quebec<br>n (%)
1	9,104 (87)	15,664 (79)
2	1,022 (10)	3,011 (15)
3	171 (2)	813 (4)
4 or more	38 (1)	417 (2)

© 2026 Meinert FM et al. *JAMA Network Open*.

eTable 6. Distribution of Types of Nongastrointestinal Bleeding in the Study Cohort

**Type of non-gastrointestinal bleeding***	**UK**<br>**n (%)**	**Quebec**<br>**n (%)**
Hematuria	19 (32)	50 (32)
Epistaxis	12 (20)	13 (8)
Bleeding as a complication from an intervention	9 (15)	35 (22)
Hemoptysis	7 (12)	10 (6)
Other nongastrointestinal bleedings	13 (18)	50 (32)

* Types of nongastrointestinal bleedings are not mutually exclusive.

© 2026 Meinert FM et al. *JAMA Network Open*.

eTable 7. Risk of GI Bleeding Associated With Concomitant Use of DOACs and COX-2–Selective NSAIDs vs Concomitant use of DOACs and Nonselective NSAIDs Among Patients With NVAF (Stratification by Demographics)

	N Patients	N Events	N PY	IR* (95%CI)	Crude HR (95%CI)	IPTW HR (95%CI)	Pooled HR (95% CI)	I^2
**<75 years**							0.58 (0.35-0.96)	0%
**UK**								
DOACs + COX-2 selective NSAIDs	928	S	S	12.70 (3.52-33.89)	0.91 (0.25-3.24)	0.93 (0.26-1.36)		
DOACs + non-selective NSAIDs	6,203	15	902	16.62 (8.21-25.04)	1.00 (reference)	1.00 (reference)		
**Quebec**								
DOACs + COX-2 selective NSAIDs	8,452	26	1,431	18.17 (11.18-25.15)	0.51 (0.30-0.88)	0.53 (0.31-0.92)		
DOACs + non-selective NSAIDs	7,045	29	725	39.98 (25.43-54.53)	1.00 (reference)	1.00 (reference)		
**≥75 years**							0.65 (0.42-0.99)	37%
**UK**								
DOACs + COX-2 selective NSAIDs	638	S	S	32.32 (12.26-70.85)	0.58 (0.23-1.51)	0.98 (0.45-2.11)		
DOACs + non-selective NSAIDs	4,055	36	565	63.77 (42.94-84.60)	1.00 (reference)	1.00 (reference)		
**Quebec**								
DOACs + COX-2 selective NSAIDs	7,087	44	1,158	37.99 (26.77-49.22)	0.53 (0.32-0.88)	0.54 (0.33-0.90)		
DOACs + non-selective NSAIDs	3,425	26	322	80.82 (49.75-111.89)	1.00 (reference)	1.00 (reference)		
**Female sex**							0.50 (0.31-0.80)	0%
**UK**								
DOACs + COX-2 selective NSAIDs	689	S	S	15.13 (4.19-40.36)	0.43 (0.13-1.48)	0.52 (0.16-1.68)		
DOACs + non-selective NSAIDs	3,898	24	552	43.51 (26.10-60.91)	1.00 (reference)	1.00 (reference)		
**Quebec**								
DOACs + COX-2 selective NSAIDs	7,677	36	1,299	27.72 (18.66-36.77)	0.48 (0.29-0.80)	0.50 (0.30-0.83)		
DOACs + non-selective NSAIDs	4,110	28	415	67.44 (42.46-92.42)	1.00 (reference)	1.00 (reference)		
**Male sex**							0.85 (0.55-1.32)	78%
**UK**								
DOACs + COX-2 selective NSAIDs	877	S	S	25.97 (9.85-56.9)	0.98 (0.37-2.59)	1.74 (0.79-3.83)		
DOACs + non-selective NSAIDs	6,360	27	915	29.50 (18.38-40.63)	1.00 (reference)	1.00 (reference)		
**Quebec**								

© 2026 Meinert FM et al. *JAMA Network Open*.

	N Patients	N Events	N PY	IR* (95%CI)	Crude HR (95%CI)	IPTW HR (95%CI)	Pooled HR (95% CI)	I^2
DOACs + COX-2 selective NSAIDs	7,862	34	1,290	26.35 (17.49-35.21)	0.67 (0.40-1.13)	0.63 (0.37-1.06)		
DOACs + non-selective NSAIDs	6,360	27	632	42.72 (26.61-58.84)	1.00 (reference)	1.00 (reference)		

S = Data suppressed due to small cells.
* IR per 1,000 PY.

Abbreviations: GI, gastrointestinal; DOACs, direct oral anticoagulants; NSAIDs, non steroidal anti-inflammatory drugs; COX-2, cyclooxygenase 2; NVAF, non-valvular atrial fibrillation; PY, patient-years; IR, incidence rate; HR, hazard ratio; CI, confidence interval; IPTW, inverse probability of treatment weighting.

© 2026 Meinert FM et al. *JAMA Network Open*.

eTable 8. Risk of GI Bleeding With Concomitant Use of DOACs and COX-2–Selective NSAIDs vs Concomitant use of DOACs and Nonselective NSAIDs Among Patients With NVAF (Stratification by User Type, Baseline Bleeding Risk, and Individual DOACs)

	N Patients	N Events	N PY	IR* (95%CI)	Crude HR (95%CI)	IPTW HR (95%CI)	Pooled HR (95% CI)	I^2
**Prevalent NSAID users or co-initiators**							0.66 (0.29-1.48)	2%
**UK**								
DOACs + COX-2 selective NSAIDs	550	S	S	10.76 (2.15-34.49)	0.36 (0.08-1.58)	0.33 (0.07-1.60)		
DOACs + non-selective NSAIDs	2,347	14	425	32.97 (15.70-50.24)	1.00 (reference)	1.00 (reference)		
**Quebec**								
DOACs + COX-2 selective NSAIDs	2,214	17	680	25.01 (13.12-36.90)	1.03 (0.37-2.87)	0.85 (0.33-2.16)		
DOACs + non-selective NSAIDs	1,111	5	164	30.47 (11.56-66.80)	1.00 (reference)	1.00 (reference)		
**Prevalent DOAC users**							0.62 (0.44-0.89)	80%
**UK**								
DOACs + COX-2 selective NSAIDs	1,016	S	S	29.27 (5.85-52.69)	0.99 (0.41-2.37)	1.37 (0.63-2.96)		
DOACs + non-selective NSAIDs	7,911	37	1,042	35.51 (24.06-46.95)	1.00 (reference)	1.00 (reference)		
**Quebec**								
DOACs + COX-2 selective NSAIDs	13,325	53	1,909	27.76 (20.28-35.23)	0.53 (0.36-0.78)	0.51 (0.34-0.75)		
DOACs + non-selective NSAIDs	9,359	50	883	56.62 (40.93-72.32)	1.00 (reference)	1.00 (reference)		
**HAS-BLED score 0-2**							0.56 (0.35-0.90)	77%
**UK**								
DOACs + COX-2 selective NSAIDs	436	S	S	23.58 (4.70-75.58)	1.45 (0.32-6.67)	2.14 (0.56-8.19)		
DOACs + non-selective NSAIDs	3,373	10	465	21.48 (8.17-34.80)	1.00 (reference)	1.00 (reference)		
**Quebec**								
DOACs + COX-2 selective NSAIDs	10,411	32	1,675	19.10 (12.49-25.72)	0.52 (0.31-0.86)	0.46 (0.28-0.77)		
DOACs + non-selective NSAIDs	7,255	31	712	43.54 (28.22-58.87)	1.00 (reference)	1.00 (reference)		
**HAS-BLED score ≥3**							0.73 (0.47-1.14)	0%
**UK**								
DOACs + COX-2 selective NSAIDs	1,130	S	S	19.61 (3.92-35.29)	0.55 (0.23-1.31)	0.82 (0.39-1.75)		
DOACs + non-selective NSAIDs	6,885	41	1,001	40.95 (28.41-53.48)	1.00 (reference)	1.00 (reference)		
**Quebec**								
DOACs + COX-2 selective NSAIDs	5,128	38	914	41.57 (28.35-54.79)	0.64 (0.38-1.08)	0.69 (0.40-1.18)		

© 2026 Meinert FM et al. *JAMA Network Open*.

	N Patients	N Events	N PY	IR* (95%CI)	Crude HR (95%CI)	IPTW HR (95%CI)	Pooled HR (95% CI)	I^2
DOACs + non-selective NSAIDs	3,215	24	335	71.60 (42.95-100.24)	1.00 (reference)	1.00 (reference)		
**Apixaban**							0.38 (0.21-0.70)	64%
**UK**								
Apixaban + COX-2 selective NSAIDs	601	S	S	12.32 (2.46-39.50)	0.65 (0.15-2.89)	0.99 (0.28-3.54)		
Apixaban + non-selective NSAIDs	3,972	15	570	26.34 (13.01-39.67)	1.00 (reference)	1.00 (reference)		
**Quebec**								
Apixaban + COX-2 selective NSAIDs	5,556	15	853	17.59 (8.69-26.49)	0.30 (0.15-0.58)	0.29 (0.15-0.58)		
Apixaban + non-selective NSAIDs	3,803	22	359	61.31 (35.69-86.93)	1.00 (reference)	1.00 (reference)		
**Rivaroxaban**							0.62 (0.38-0.99)	47%
**UK**								
Rivaroxaban + COX-2 selective NSAIDs	709	6	167	35.86 (7.17-64.55)	0.97 (0.40-2.39)	1.05 (0.43-2.58)		
Rivaroxaban + non-selective NSAIDs	4,798	28	687	40.77 (25.67-55.88)	1.00 (reference)	1.00 (reference)		
**Quebec**								
Rivaroxaban + COX-2 selective NSAIDs	6,319	28	1,029	27.22 (17.14-37.30)	0.57 (0.33-1.01)	0.50 (0.29-0.88)		
Rivaroxaban + non-selective NSAIDs	4,387	23	439	52.45 (31.01-73.88)	1.00 (reference)	1.00 (reference)		

S = Data suppressed due to small cells.
* IR per 1,000 PY.

Abbreviations: GI, gastrointestinal; DOACs, direct oral anticoagulants; NSAIDs, non steroidal anti-inflammatory drugs; COX-2, cyclooxygenase 2; NVAF, non-valvular atrial fibrillation; PY, patient-years; IR, incidence rate; HR, hazard ratio; CI, confidence interval; IPTW, inverse-probability-of-treatment-weighting.

© 2026 Meinert FM et al. *JAMA Network Open*.

eTable 9. Risk of GI Bleeding With Concomitant Use of DOACs and COX-2–Selective NSAIDs vs Concomitant use of DOACs and Nonselective NSAIDs Among Patients With NVAF (Sensitivity Analyses)

	N Patients	N Events	PY	IR* (95%CI)	Crude HR (95%CI)	IPTW HR (95%CI)	Pooled HR (95% CI)	I^2
**15-day grace period**							0.64 (0.46-0.89)	72%
**UK**								
DOACs + COX-2 selective NSAIDs	1,566	8	355	22.52 (6.92-38.13)	0.80 (0.37-1.71)	1.14 (0.58-2.23)		
DOACs + non-selective NSAIDs	10,258	47	1,397	33.64 (24.02-43.26)	1.00 (reference)	1.00 (reference)		
**Quebec**								
DOACs + COX-2 selective NSAIDs	15,539	64	2,497	25.68 (19.39-31.97)	0.58 (0.40-0.84)	0.54 (0.37-0.78)		
DOACs + non-selective NSAIDs	10,470	53	1,027	51.58 (37.69-65.47)	1.00 (reference)	1.00 (reference)		
**Stricter outcome definition**							0.83 (0.51-1.34)	58%
**UK**								
DOACs + COX-2 selective NSAIDs	1,566	6	391	15.35 (3.07-27.63)	1.01 (0.41-2.49)	1.38 (0.61-3.10)		
DOACs + non-selective NSAIDs	10,258	27	1,468	18.39 (11.45-25.32)	1.00 (reference)	1.00 (reference)		
**Quebec**								
DOACs + COX-2 selective NSAIDs	15,539	27	2,593	10.41 (6.49-14.34)	0.70 (0.38-1.30)	0.62 (0.34-1.14)		
DOACs + non-selective NSAIDs	10,470	18	1,050	17.14 (9.22-25.07)	1.00 (reference)	1.00 (reference)		
**Inclusion of fatal events**							0.70 (0.49-0.99)	34%
**UK**								
DOACs + COX-2 selective NSAIDs	1,566	8	391	20.47 (6.28-34.65)	0.69 (0.32-1.48)	0.998 (0.51-1.95)		
DOACs + non-selective NSAIDs	10,258	51	1,467	34.77 (25.23-44.31)	1.00 (reference)	1.00 (reference)		
**Quebec******								
DOACs + COX-2 selective NSAIDs	11,377	58	1,948	29.78 (22.11-37.44)	0.64 (0.43-0.97)	0.61 (0.40-0.92)		
DOACs + non-selective NSAIDs	7,455	41	760	53.92 (37.41-70.42)	1.00 (reference)	1.00 (reference)		
**Exclusion of prior events**							0.65 (0.45-0.95)	0%
**UK**								
DOACs + COX-2 selective NSAIDs	1,504	6	374	16.03 (3.20-28.85)	0.59 (0.25-1.40)	0.91(0.43-1.92)		
DOACs + non-selective NSAIDs	9,942	44	1,429	30.78 (21.69-39.88)	1.00 (reference)	1.00 (reference)		
**Quebec**								
DOACs + COX-2 selective NSAIDs	13,750	54	2,323	23.25 (17.05-29.45)	0.66 (0.43-1.01)	0.59 (0.39-0.90)		

© 2026 Meinert FM et al. *JAMA Network Open*.

	N Patients	N Events	PY	IR* (95%CI)	Crude HR (95%CI)	IPTW HR (95%CI)	Pooled HR (95% CI)	I^2
DOACs + non selective NSAIDs	9,122	38	927	40.98 (27.95-54.00)	1.00 (reference)	1.00 (reference)		
**Multiple imputation*** **							NA	
**UK**								
DOACs + COX-2 selective NSAIDs	1,556	8	391	20.47 (6.28-34.65)	0.69 (0.32-1.48)	0.93 (0.47-1.85)		
DOACs + non-selective NSAIDs	10,258	51	1,467	34.77 (25.23-44.31)	1.00 (reference)	1.00 (reference)		
**One treatment episode only**							0.61 (0.43-0.86)	50%
**UK**								
DOACs + COX-2 selective NSAIDs	1,368	7	361	19.36 (5.02-33.71)	0.60 (0.27-1.35)	0.94 (0.47-1.88)		
DOACs + non-selective NSAIDs	8,967	50	1,309	38.20 (27.61-48.79)	1.00 (reference)	1.00 (reference)		
**Quebec**								
DOACs + COX-2 selective NSAIDs	11,726	58	2,078	27.91 (20.72-35.09)	0.56 (0.38-0.83)	0.53 (0.36-0.79)		
DOACs + non-selective NSAIDs	8,179	47	825	56.95 (40.67-73.23)	1.00 (reference)	1.00 (reference)		
**Excluding prior GI conditions********							0.58 (0.40-0.84)	58%
**UK**								
DOACs + COX-2 selective NSAIDs	1,326	7	328	21.34 (5.53-37.15)	0.76 (0.34-1.72)	0.98 (0.46-2.11)		
DOACs + non-selective NSAIDs	8,836	41	1,265	32.42 (22.49-42.34)	1.00 (reference)	1.00 (reference)		
**Quebec**								
DOACs + COX-2 selective NSAIDs	13,461	50	2,268	22.05 (15.94-28.16)	0.55 (0.36-0.83)	0.49 (0.32-0.76)		
DOACs + non-selective NSAIDs	9,095	41	921	44.53 (30.90-58.17)	1.00 (reference)	1.00 (reference)		

* IR per 1,000 PY.
** The study period for this analysis ended on December 31, 2018 due to lack of availability of cause of death in the Quebec data source beyond this date.
*** This analysis was conducted only in the UK data source for missing values for body mass index and blood pressure.
**** GI conditions included enteritis, colitis, or ulcer.

Abbreviations: GI, gastrointestinal; DOACs, direct oral anticoagulants; NSAIDs, non-steroidal anti-inflammatory drugs; COX-2, cyclooxygenase 2, NVAF, non-valvular atrial fibrillation; PY, patient-years; IR, incidence rate; HR, hazard ratio; CI, confidence interval; NA, not applicable.

© 2026 Meinert FM et al. *JAMA Network Open*.
