# Supplementary material for: Direct Oral Anticoagulants, COX-2–Selective NSAIDs, and Gastrointestinal Bleeding in Atrial Fibrillation
Source: JAMA Netw Open. 2026 May 26;9(5):e2613941. doi: 10.1001/jamanetworkopen.2026.13941 (PMC13213523; doi:10.1001/jamanetworkopen.2026.13941)
Supplement: Supplement 2. — Data Sharing Statement [file jamanetwopen-e2613941-s002.pdf]

Data Sharing Statement

Data
Data available: No

Additional Information
Explanation for why data not available: The data for this study are available from the data custodians of the UK CPRD and the RAMQ. Restrictions apply to the availability of these data, which were used under license for this study.
